# Correlation of anterior cerebral artery resistive index with early comorbidities in preterm neonates

**DOI:** 10.3389/fped.2024.1441553

**Published:** 2024-09-12

**Authors:** Karambir Singh Gill, Bhavna Gupta, Puneet A. Pooni, Siddharth Bhargava

**Affiliations:** Department of Pediatrics, Dayanand Medical College and Hospital, Ludhiana, India

**Keywords:** RI, ACA, preterm, neonates, Doppler RI, Doppler

## Abstract

**Introduction:**

This study was undertaken to find the clinical correlation of resistive index (RI) in the anterior cerebral artery (ACA) of preterm neonates admitted to the Neonatal Intensive care unit (NICU) with comorbidities such as perinatal asphyxia, neonatal sepsis, and necrotizing enterocolitis (NEC).

**Methods:**

An observational analytical study was conducted, including preterm neonates (<35 weeks) admitted to the NICU. Ultrasound cranium scans were performed on days 1–3 and 7 of life as per the study protocol. Baseline and clinical data of asphyxia, sepsis, and NEC were obtained. Images were acquired using a 4–8-MHz probe on a Sonosite M-turbo machine (Bothell, WA, USA). All statistical calculations were done using SPSS version 21.0 (SPSS Inc., Chicago, IL, USA) with the application of the Kolmogorov–Smirnov test and the Mann–Whitney test.

**Results:**

During the study period, a total of 739 neonates were admitted. Of these, 73 neonates constituted the study group. Among the 73 patients, 33 were preterm neonates without comorbidities and 40 neonates had comorbidities such as perinatal asphyxia, sepsis, and NEC stage 2 and 3 (necrotizing enterocolitis). In the present study, the mean RI on day 3 of life was 0.76 ± 0.04 in neonates without comorbidities and 0.77 ± 0.04 in neonates with comorbidities, with a *p*-value of 0.247. On the 7th day of life, the mean RI was 0.82 ± 0.03 in both groups, with a *p*-value of 0.42.

**Conclusion:**

We could not find any significant clinical correlation of RI in the ACA of preterm neonates <35 weeks of gestation with comorbidities.

## Introduction

Neonatal cranial ultrasound is a non-invasive, bedside-available modality useful for neonatologists in intensive care units. It is part of the point-of-care ultrasound performed by neonatologists, which helps in early diagnosis and timely management. In preterm neonates, there is an increased tendency for acquired brain injury during the perinatal period, which can lead to long-term neurodevelopmental consequences (1). Preterm neonates have pressure-passive circulation because of immature auto-regulation of cerebral circulation. Any hemodynamic instability in systemic perfusion directly affects cerebral perfusion (2). Changes in cerebral perfusion pressure play an important role in the pathogenesis of preterm brain injury (3). Many factors are responsible for altered cerebral hemodynamics in preterm babies, including sepsis, shock, perinatal asphyxia, necrotizing enterocolitis (NEC), and respiratory failure requiring mechanical ventilation (4). Very few treatment options are available for preterm brain injury, so our focus is on the prevention of the same. Long-term consequences of preterm brain injury include neurodevelopmental (cerebral palsy), cardiovascular, endocrine, and metabolic disorders (5–7). Cerebral hemodynamic monitoring may help in the early detection of altered cerebral perfusion pressure and guide early intervention. Low cerebral oxygenation in preterms has been associated with poor neurodevelopmental outcomes (8).

Using bedside neonatal cranial ultrasound with color Doppler imaging, the resistive index (RI) of cerebral arteries can be calculated. This non-invasive modality is safe and can be performed at the bedside (9). Term neonates with birth asphyxia have a low resistive index. Ultrasound Doppler using anterior cerebral artery (ACA) RI can be used to diagnose perinatal asphyxia in resource-limited facilities (10). Preterm neonates with hemodynamically significant patent ductus arteriosus have a high resistive index (11, 12). The resistive index can be measured serially in the same vessel of a neonate for early detection of changes in cerebral hemodynamics associated with the onset of comorbidities. RI may vary depending on the nature of the vessel targeted for measurement (13). In our study, we compared the values of the resistive index in the anterior cerebral artery of healthy preterm neonates with those of preterm neonates who have comorbidities.

## Aims and objectives

This study aimed to assess the clinical correlation of ACA RI with early comorbidities in preterm neonates admitted to Neonatal Intensive care unit (NICU)s.

## Materials and methods

This prospective observational study was conducted in the Neonatal Intensive Care Unit of Dayanand Medical College and Hospital, Ludhiana (Punjab), over a period of 12 months from January to December 2022. As per the inclusion criteria, all inborn preterm babies with a birth gestation of ≤35 weeks admitted to the NICU were included in the study. As per the NRP (Neonatal Resuscitation Program) guidelines, 35 weeks is the cutoff for birth gestation to classify neonates into preterm and term for initial oxygen use during neonatal resuscitation at birth.

The exclusion criteria included the following: (a) birth gestation >35 weeks; (b) loss to follow-up; (c) congenital malformations; and (d) refusal to participate in the study by the parent/guardian.

Based on the incidence of preterm births and considering the level of significance at 5%, the sample size was calculated as 70. Informed consent was obtained on the consent/accent form from the parents of all study subjects, and the study was approved by the institutional ethics committee (IEC) DMCH under number DMCH/IEC/2023/209 in accordance with the Declaration of Helsinki. Mother's antenatal details, including age, gravida, parity status, past obstetric history, history of antenatal visits, antenatal complications, antenatal ultrasound findings, and antenatal Doppler images, were recorded as per a structured pro forma. Birth details, including birth gestation, birth weight, type of delivery, and Apgar score, were recorded to identify neonates with perinatal asphyxia.

During the postnatal stay in the NICU, babies were categorized into two groups: preterm neonates without comorbidities and those with comorbidities such as perinatal asphyxia, sepsis, and NEC. Perinatal asphyxia was defined as neonates who did not cry at birth as per the National Neonatal Perinatal Database (NNPD). Based on the Apgar score, moderate perinatal asphyxia was defined as an Apgar score of 4–6, while severe asphyxia was defined as no breathing or an Apgar score of 0–3 at 1 min of life. Sepsis was defined as symptomatic neonates with positive sepsis markers, with or without a positive blood culture. Necrotizing enterocolitis was defined as neonates with feed intolerance, abdominal distension, or intestinal perforation, classified as per Bell's staging. Postnatal details, including the need for continuous positive airway pressure (CPAP), surfactant, mechanical ventilation, inotropes, and primary diagnosis with outcome, were noted as per a structured pro forma.

All neonates underwent cranial ultrasound scanning along with color Doppler imaging as per the screening protocol. The first examination was done within 72 h of NICU admission, preferably at 24 h of life, followed by a second examination on days 7–10 of life. For babies with prolonged NICU stay, a day 14 scan was also conducted. Images were acquired in the sagittal plane through the anterior fontanel using a 4–8-MHz probe on a Sonosite M-turbo machine (Bothell, WA, USA). In the sagittal plane, the ACA was visualized using color flow mode, followed by pulsed wave Doppler flow assessment of blood flow in the ACA to calculate the RI. Three readings were taken for every scan. RI was calculated from Doppler waveforms of the ACA, with a caliper placed in the sagittal plane. Vmax and RI readings were obtained and noted.

The resistive index is a non-invasive method for assessment of cerebral blood flow (5). It is defined as follows (14):

$$\mathrm{Resistive}\;\mathrm{index}\;=\;\frac{\mathrm{Peak}\;\mathrm{systolic}\;\mathrm{velocity}-\mathrm{Diastolic}\;\mathrm{velocity}}{\mathrm{Peak}\;\mathrm{systolic}\;\mathrm{velocity}}$$

Data are described in terms of range, mean ± standard deviation (SD), median (IQR), frequencies (number of cases), and relative frequencies (percentages) as appropriate. The Kolmogorov–Smirnov test was used to determine whether the data were normally distributed. A comparison of quantitative variables between the study groups was performed using the Mann­–Whitney *U-*test and the Kruskal–Wallis test for non-parametric data. For comparing categorical data, the chi-square (χ^2^) test was performed, and the Fisher exact test was used when the expected frequency was less than 5. The receiver operator characteristic (ROC) curve was used to estimate the criterion value depending on the specificity and sensitivity, and the area under the curve (AUC) was measured. A probability value (*p*-value) less than 0.05 was considered statistically significant. All statistical calculations were performed using Statistical Package for the Social Science version 21.0 (SPSS) (SPSS Inc., Chicago, IL, USA) for Microsoft Windows.

### Characteristics of the study group

**Figure 1:**
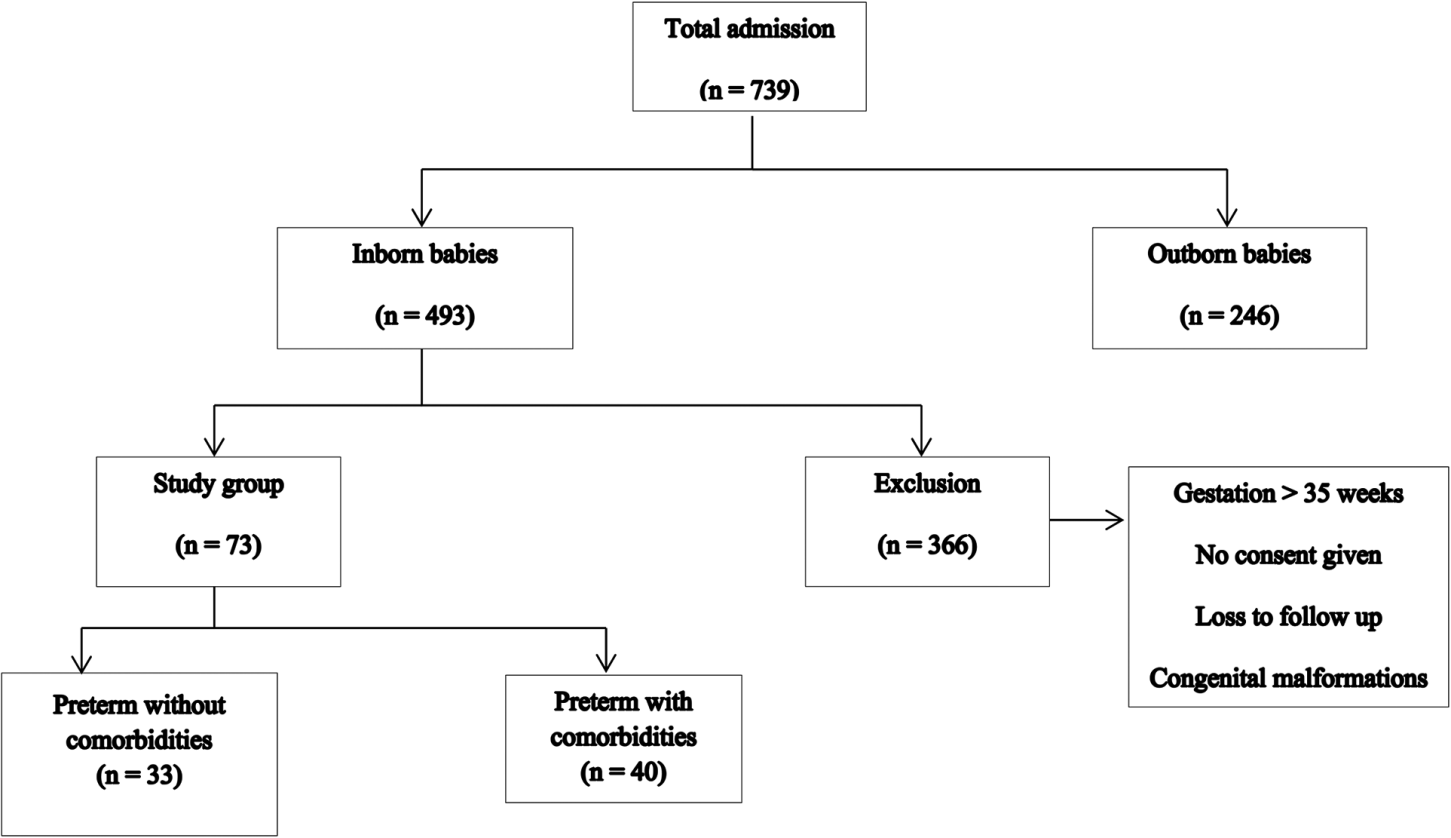
Characteristics of the study group.

The study group characteristics are shown in Figure 1.

## Results

During the study period, there were a total of 739 intramural and extramural admissions, with 493 being intramural admissions. Among these, 73 infants constituted the study group. Of these 73 patients, 33 were preterm neonates without comorbidities, while 40 neonates had other comorbidities such as perinatal asphyxia, sepsis, and NEC stage 2 and 3 (Table 1).

**Table 1:** Maternal and neonatal demographics.

S. no.	Variables	Statistics, *n* (%)
1	Total subjects	73
2	Gestation	
	<28 weeks	12
	28–32 weeks	36
	32–35 weeks	25
3	Gender	
	Male	47 (64%)
	Female	26 (36%)
4	Antenatal steroids	49
5	SGA	11
6	Mode of delivery	
	LSCS	66
	Vaginal delivery	7
7	Mean gestation (range)	30 + 4 weeks (25–35 weeks)
8	Mean birth weight	1,383 g
9	Apgar score at 1 min	
	Less than 3	3
	4–6	13
	Apgar score at 5 min	
	Less than 3	2
	4–6	2
10	CPAP	62 (85%)
11	Surfactant	17 (23%)
12	Mechanical ventilation	12 (16%)
13	Inotropes	11 (15%)
14	Asphyxia	16 (22%)
15	Sepsis	15 (20%)
16	NEC	9 (12.3%)
17	Mean duration of NICU stay (range)	26 days (10–60 days)
18	Outcome	
	Discharged	66 (90.5%)
	Died	4 (5.5%)
	Discharged against medical advice	3 (4%)
19	Primary diagnosis	
	• RDS • Perinatal asphyxia • Sepsis • NEC	22 (30%) 16 (22%) 15 (20%) 9 (12.3%)
	Stage 2–5	
	Stage 3–4	
	• NEC + sepsis • Others	6 (8.2%) 6 (8.2%)

The most common reason for admission to NICU was respiratory distress syndrome (RDS), with 62 (85%) babies requiring CPAP and 17 (23%) requiring surfactant. Among the 73 patients, 16 (22%) neonates had perinatal asphyxia at birth and required neonatal resuscitation, while 12 (16%) were mechanically ventilated. Antenatal steroids were administered to 49 patients. Out of these 73 neonates, 11 were classified as small for gestational age (SGA). Apgar scores of less than 3 (severe perinatal asphyxia) at 1 min of age were recorded in 3 neonates, and moderate asphyxia was observed in 13 babies. There were 15 (20%) neonates with clinical sepsis and positive sepsis markers, with 11 (15%) requiring inotropes and 9 having NEC. Of the 11 babies with hypotensive shock requiring inotropic support, only 4 survived. Six preterm babies had both NEC and sepsis. Intraventricular hemorrhage (IVH) was seen in six babies, with IVH grade I in four and IVH grade II in two. Patent ductus arteriosus (PDA) was detected in 17 preterm babies, of which 7 were found to be hemodynamically significant and required medical management. Mean Hb was 16 (SD 2). Anemia (Hb <11 gm/dl) was seen in two babies at admission. As per the outcome, 66 (90.5%) babies were discharged, with a mean duration of NICU stay of 28 days (Table 2).

**Table 2:** Values of the mean RI in the ACA.

	GA (weeks)	*n*	Mean	Std. deviation	Median	IQR	*F*	*p*-value
RI (3D)	<28	12	0.78	0.03	0.79	0.7425–0.82	2.928	0.231
	28.1–32	36	0.76	0.05	0.75	0.7325–0.78		
	32.1–35	25	0.76	0.04	0.76	0.73–0.79		
RI (7D)	<28	12	0.82	0.04	0.83	0.7775–0.84	0.381	0.826
	28.1–32	36	0.82	0.03	0.83	0.8025–0.84		
	32.1–35	25	0.83	0.03	0.82	0.8–0.85		
** **	Weight (kg)	*n*	Mean	Std. deviation	Median	IQR	*F*	*p*-value
RI (3D)	<1	21	0.79	0.04	0.79	0.75–0.82	11.52	0.003
	1–1.5	19	0.75	0.05	0.74	0.72–0.77		
	1.5–2.5	33	0.76	0.04	0.76	0.735–0.785		
RI (7D)	<1	21	0.84	0.03	0.83	0.815–0.86	7.626	0.022
	1–1.5	19	0.81	0.04	0.81	0.77–0.83		
	1.5–2.5	33	0.82	0.03	0.82	0.8–0.845		

In this study, we calculated the mean RI on days 3 and 7 of life for preterm babies. Newborns without comorbidities were divided into various groups based on gestational age and weight. The mean RI was measured separately for each group on days 3 and 7 of life. For gestational age, the mean RI was 0.78 ± 0.03 (≤28 weeks), 0.76 ± 0.04 (28 + 1 to 32 weeks), and 0.76 ± 0.04 (32 + 1 to 35 weeks) on day 3 of life and 0.82 ± 0.04 (≤28 weeks), 0.82 ± 0.03 (28 + 1 to 32 weeks), and 0.83 ± 0.03 (32 + 1 to 35 weeks) on day 7 of life. For weight, the mean RI was 0.79 ± 0.04 (≤1,000 g), 0.75 ± 0.05 (1,001–1,500 g), and 0.76 ± 0.04 (1,501–2,500 g) on day 3 of life and 0.85 ± 0.03 (≤1,000 g), 0.81 ± 0.03 (1,001–1,500 g), and 0.82 ± 0.02 (1,501–2,500 g) on day 7 of life. The ACA RI on day 3 increased as gestational age decreased. Additionally, the ACA RI on days 3 and 7 was higher in babies weighing less than 1 kg. As birth weight increased, the RI decreased, and this relationship was found to be statistically significant (Table 3).

**Table 3:** Variability of the mean RI with comorbidities.

		*n*	Mean	SD	Median	IQR	*Z*	*p*-value
RI (3D)	Neonates without comorbidities	33	0.76	0.04	0.75	0.73–0.785	−1.157	0.247
	Neonates with comorbidities	40	0.77	0.04	0.77	0.74–0.80		
RI (7D)	Neonates without comorbidities	33	0.83	0.03	0.83	0.805–0.85	−0.803	0.422
	Neonates with comorbidities	40	0.82	0.03	0.83	0.8–0.84		

In the present study, the mean RI on day 3 of life was 0.76 ± 0.04 in newborns without comorbidities and 0.77 ± 0.04 in newborns with comorbidities, with a *p*-value of 0.247. On day 10 of life, the mean RI was 0.83 ± 0.03 in both groups, with a *p*-value of 0.42. A ROC curve was created, which demonstrated a cutoff for ACA RI on day 3 of >0.82 for the prediction of mortality with an AUC of 0.645. For an ACA RI >0.82, the sensitivity is 43%, while the specificity is 88% (Table 4).

**Table 4:** Variability of the mean RI with perinatal asphyxia, sepsis, and NEC.

		*n*	Mean	SD	Median	IQR	*F*	*p*-value	*p*-value
RI (3D)	NO	33	0.76	0.04	0.75	0.73–0.785	1.805	0.138	
	Asphyxia	16	0.76	0.04	0.76	0.74–0.79			0.998
	Sepsis	9	0.77	0.04	0.77	0.745–0.805			0.927
	NEC	9	0.75	0.03	0.75	0.735–0.77			0.994
	Sepsis + NEC	6	0.81	0.05	0.81	0.7625–0.85			0.108
RI (7D)	NO	33	0.83	0.03	0.83	0.805–0.85	0.625	0.646	
	Asphyxia	16	0.82	0.04	0.83	0.8–0.8475			0.973
	Sepsis	9	0.82	0.03	0.83	0.795–0.835			0.935
	NEC	9	0.81	0.03	0.81	0.795–0.83			0.828
	Sepsis + NEC	6	0.84	0.03	0.84	0.8–0.8725			0.965

NO, preterms without comorbidity.

In our study, neonates with perinatal asphyxia had a mean RI of 0.76 ± 0.04 on day of life 3 (*p*-value 0.9) and 0.82 ± 0.04 on day 10 of life (*p*-value 0.9). Neonates with sepsis had a mean RI of 0.78 ± 0.04 on day 3 of life (*p*-value 0.9) and 0.82 ± 0.02 on day 10 of life (*p*-value 0.83). Neonates with NEC had a mean RI of 0.75 ± 0.04 on day 3 of life (*p*-value 0.9) and 0.82 ± 0.03 on day 10 of life (*p*-value 0.68) (Table 5).

**Table 5:** Mean RI on day 3 vs. day 7.

	RI (3D)	RI (7D)	*z*	*p*-value
Mean	0.77	0.82	−6.637	0.001
SD	0.04	0.03		
Median	0.76	0.83		
IQR	0.74–0.79	0.80–0.84		

**Figure 2:**
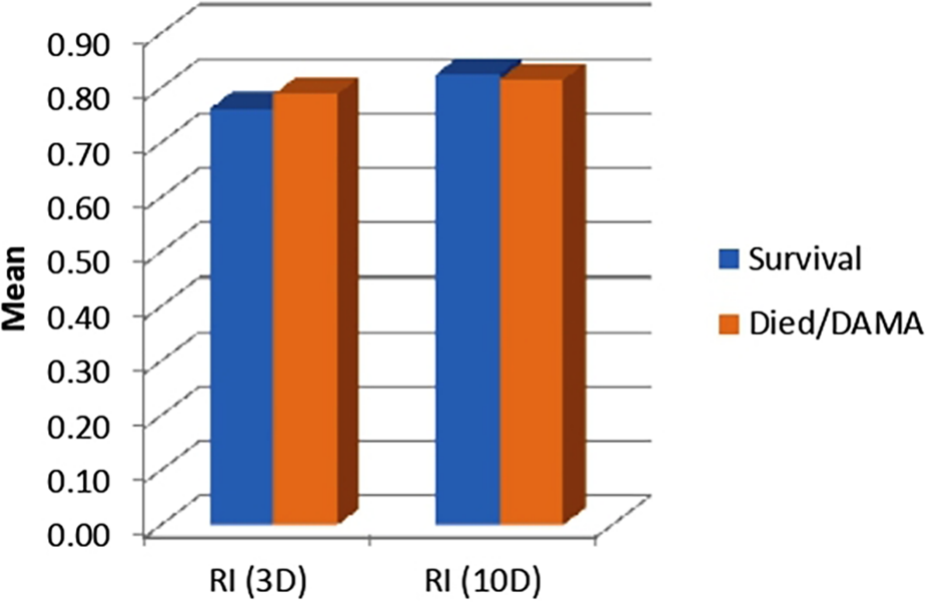
RI—survival vs. mortality group.

The mean RI increased from 0.77 ± 0.04 on day 3 to 0.82 ± 0.03 on day 7, which is statistically significant. This indicated that RI increases with the increase in chronological age (Figure 2).

The mean RI on day 3 was 0.77 ± 0.04 in survivors and 0.79 ± 0.05 (*p* = 0.208) in non-survivors. The mean RI on day 7 was 0.82 ± 0.03 in survivors and 0.82 ± 0.03 in non-survivors (*p* = 0.376). Both the *p*-values were found to be non-significant.

## Discussion

In preterm neonates, cerebral hemodynamic monitoring can be used as a preventive tool for acquired preterm brain injury (9). The resistive index of cerebral arteries can be used as a measure of cerebral hemodynamics (9). Risk factors for preterm brain injury cause altered cerebral perfusion pressure because of pressure-passive circulation, which is considered a major pathophysiological mechanism underlying brain injury (2). For timely detection of increased or decreased resistive index of cerebral arteries in preterm babies, we need to define reference values specific to the Indian context. Then, we need to compare the variability of RI values between healthy preterm neonates and those with comorbidities.

In this study, we observed that ACA RI values in healthy preterm neonates show a rising trend from day 3 to day 7 of life. Calvert et al. (11) assessed RI during the first 72 h of life for babies with gestations of 26–28 and 29–32 weeks. Their values contrast with our results. On the other hand, RI values assessed during the first 8 h of life by Pezzati et al. (15) correlate with our findings. RI values assessed by Romagnoli et al. (16) on days 1, 3, 7, 14, 21, and 28 of life for various gestational age groups did not show the same rising trend as observed in our study, but their values on day 3 of life correlate with our values.

In our study, the ACA RI on day 3 increased as gestational age decreased, but this observation was not statistically significant. The ACA RI on days 3 and 7 was higher in babies weighing less than 1 kg. As birth weight increased, the RI decreased, which was statistically significant (*p* < 0.05).

In a recent study, it was observed that the resistive index is higher in large arteries and lower in smaller arteries. Thus, for comparison purposes, we need to compare the RI of the same arteries across all babies. However, no difference was found between the RI values of left- and right-sided arteries of the same baby (12).

In this study, we examined the preterm babies to calculate ACA RI values as a marker for assessment of cerebral perfusion and compared the RI values in preterm babies without comorbidities with those in preterm babies with comorbidities such as asphyxia, sepsis, and NEC.

Term neonates with perinatal asphyxia have a low RI in the initial stages because of cerebral vasodilation and increased cerebral diastolic blood flow, but newborns with severe asphyxia have a high RI due to impaired auto-regulation and increased vascular resistance (17). Cranial ultrasound along with Doppler imaging has been proven as a useful modality to assess for cerebral perfusion and predict adverse outcomes newborns with perinatal asphyxia (18), but very little is known about its utility in preterm neonates with asphyxia.

Neonates with sepsis have impaired blood–brain barrier due to inflammation, leading to altered cerebral hemodynamics (19). Various studies have demonstrated the association of both increased and decreased cerebral blood flow in neonates with sepsis. In a recent study, increased values of RI were demonstrated in the ACA of newborns with proven sepsis, indicating decreased cerebral blood flow with sepsis (19).

We observed that RI values have no statistically significant variation between healthy preterm neonates and preterm neonates with complications like perinatal asphyxia, sepsis, and NEC. Similar results were obtained in a systematic review by Camfferman et al. (20).

Superior vena cava flow, left ventricular output, and ACA Doppler measurements have been used to predict IVH in preterm babies (21).

In a study, the ACA RI was compared with cerebral oxygen saturation detected by near-infrared spectroscopy (NIRS) in preterm neonates born before 32 weeks of gestation, paving the way for future research (22–25).

The limitations of the study include the absence of MCA (middle cerebral artery) Doppler data, lack of blood gas analysis at the time of admission, and the absence of a detailed hemodynamic assessment along with Doppler evaluation. Individual values of peak systolic velocity (PSV) and end-diastolic velocity (EDV) can be assessed along with the RI for further evaluation of cerebral perfusion. Variables affecting Doppler flow measurements, including heart rate, temperature, and anemia, need more detailed assessment in all preterm babies, as these parameters affect cerebral blood flow velocity. Integration of NIRS with other cerebral perfusion parameters could further aid in the prevention of preterm brain injury.

## Conclusion

Cerebral perfusion is important in the context of acquired preterm brain injury, and the resistive index serves as a marker for cerebral hemodynamics. However, we could not find any significant clinical correlation between ACA RI and early comorbidities in preterm neonates.

## Data Availability

The raw data supporting the conclusions of this article will be made available by the authors without undue reservation.
